# Impact of surgeon experience level on outcomes of three different root coverage procedures for RT1 gingival recession

**DOI:** 10.1186/s40902-026-00514-y

**Published:** 2026-05-29

**Authors:** Wentao Sun, Jiachen Dong, Mengjun Sun, Yue Liao, Zhongchen Song

**Affiliations:** Department of periodontology, Shanghai Ninth People′s Hospital, Shanghai Jiao Tong University School of Medicine, College of Stomatology, Shanghai Jiao Tong University, National Center for Stomatology, National Clinical Research Center for Oral Diseases, Shanghai Key Laboratory of Stomatology, Shanghai Research Institute of Stomatology, Shanghai, China

**Keywords:** Gingival recession, Root coverage procedures, Surgeon’s experience level, Coronally advanced flap, Envelope coronally advanced flap, Vestibular incision subperiosteal tunnel access

## Abstract

**Background:**

Gingival recession (GR) is a common dental condition. Root coverage procedures (RCPs) aim to restore the gingival margin to its original position and maximize root surface coverage. The impact of surgeon experience on surgical outcomes has not been systematically evaluated. This retrospective study aimed to investigate the impact of surgeon experience level on the outcomes of 3 different RCPs for RT1 GR by comprehensively assessing clinical and esthetic parameters before and after surgery.

**Results:**

A total of 304 patients (1,020 teeth) were included in this study. Surgeons with more than 5 years of experience achieved a significantly higher percentage of root coverage (PRC) (96.34 ± 10.95%) and significantly lower clinical attachment level (CAL) values (1.20 ± 0.42 mm) than less experienced surgeons (*p* < 0.05). Notably, surgical duration decreased markedly with increasing surgical experience (*p* < 0.0001). The root coverage esthetic score (RES) did not differ significantly among the three groups. Group A demonstrated higher mucosal scar index (MSI) values for the coronally advanced flap combined with connective tissue graft (CAF + CTG).

**Conclusion:**

Surgeon experience level is associated with clinical efficacy and surgical efficiency in RCPs, with more experienced surgeons achieving superior PRC and attachment gain. Esthetic outcomes were comparable across experience levels. Additionally, more experienced surgeons had shorter operative times.

## Introduction

Gingival recession (GR) frequently leads to esthetic concerns, hypersensitivity, plaque retention, and root caries [12]. Root coverage procedures (RCPs) represent a collective term for a series of periodontal plastic surgeries [19, 34]. The objective of RCPs is to position the receded gingival margin as coronally as possible toward the tooth crown and to maximize coverage of the exposed root surface. Over the years, the introduction and widespread adoption of various RCPs have prompted an increasing number of practitioners, not only periodontal surgeons, to perform surgical treatment for GR [2, 23, 33, 38, 39]. Surgeon experience can influence clinical judgment regarding surgical indications and procedural details, representing one of the operator-dependent factors affecting surgical outcomes [24, 28, 36]. The same surgical procedure may yield significantly different outcomes when performed by different surgeons. At present, numerous studies have been published on the indications and prognosis of different RCPs [5, 6, 8, 27]. Factors influencing the prognosis of RCPs can generally be categorized as patient-related, tooth-related, and treatment-related [5]. Among treatment-related factors, relatively well-defined conclusions have been reached regarding the effects of connective tissue grafts (CTGs), root surface treatment methods, and control of gingival flap tension on prognosis [7, 31, 32]. However, the impact of surgeon experience level on surgical outcomes has seldom been systematically evaluated. Researchers usually state that “the surgery is conducted by experienced operators”. However, the definition of “experienced” is rather subjective and may introduce bias. Ozcan et al. [28] evaluated the outcomes of the first 40 coronally advanced flaps (CAFs) performed by a periodontology resident and reported that surgeon experience significantly affected clinical and esthetic outcomes, surgical duration, and the incidence of complications.

Clinicians have proposed various surgical techniques to expand the indications for RCPs and improve outcomes in complex cases. In 1975, Bernimouli et al. [2] first proposed the concept of CAF in a publicly published journal. To avoid the vertical incisions associated with CAF, Zucchelli et al. [40] modified the classic CAF and subsequently introduced the envelope CAF (eCAF) technique. To increase gingival flap stability during healing, Langer and Langer [26] proposed the combined use of CTG, thereby improving the long-term stability of RCPs. To date, the combination of CAF and CTG remains the gold standard for RCPs [5]. Moreover, to minimize surgical trauma and prevent complications such as gingival papilla necrosis during healing, Zabalegui et al. [38] and Blanes et al. [3] independently proposed the tunnel technique. In 2011, Zadeh et al. [39] introduced the vestibular incision subperiosteal tunnel access (VISTA) technique, which reduces the technical difficulty of the tunnel technique by making vertical incisions in the vestibular sulcus. The outcomes of these procedures may differ depending on the surgeon’s level of surgical experience.

We hypothesized that surgeon experience level would influence clinical and esthetic outcomes of RCPs. This study conducts a comparative analysis of the surgical outcomes achieved by periodontal surgeons with varying levels of experience in performing RCPs for treating RT1 GR. Additionally, the technical sensitivity of different RCPs is evaluated. From the perspective of surgeon experience, this study provides insights for surgical strategy formulation and prognosis assessment.

## Materials and methods

### Participants

This retrospective study included patients who visited the Department of Periodontology, Ninth People’s Hospital, Shanghai Jiao Tong University School of Medicine, and underwent RCPs between January 2022 and December 2024. This study was conducted and reported following the Strengthening the Reporting of Observational Studies in Epidemiology (STROBE) reporting guidelines.

The inclusion criteria were as follows: (1) at least one tooth diagnosed with RT1 GR [18]; (2) adults aged 18–65 years; (3) no history of systemic disease or contraindications to periodontal treatment; (4) no periodontal surgery in the previous 24 months; (5) completion of comprehensive initial periodontal treatment at the time of inclusion, with full-mouth plaque percentage < 20% and full-mouth bleeding on probing scores < 20%; and (6) no calculus on teeth with GR or their adjacent teeth. The exclusion criteria were as follows: (1) patients with uncontrolled oral mucosal diseases, maxillofacial bone lesions, or other oral diseases that could affect RCP outcomes; (2) teeth with non-carious cervical lesions, endodontic diseases, or restorations; (3) pregnant or lactating women; (4) patients with severe smoking habits (> 10 cigarettes per day) or alcohol abuse; (5) patients with restricted mouth opening or unable to cooperate with treatment; and (6) patients who refused to participate.

### Group design

All periodontal surgeons who performed the operations held valid medical practitioner certificates and had completed comprehensive theoretical courses on mucogingival surgery as well as training in model-based operations. Patients were divided into three groups according to surgeon experience level at the time of surgery:

- Group A: Surgeons with ≤ 2 years of surgical experience who had performed at least 10 RCPs prior to study enrollment.
- Group B: Surgeons with > 2 years and ≤ 5 years of surgical experience who had performed at least 20 RCPs prior to study enrollment.
- Group C: Surgeons with > 5 years of surgical experience who had performed at least 50 RCPs prior to study enrollment.

Grouping was conducted according to the regional physician title system. The three groups correspond to resident (trainee), resident, and attending physician, respectively.

### Surgical procedures

**Fig. 1:**
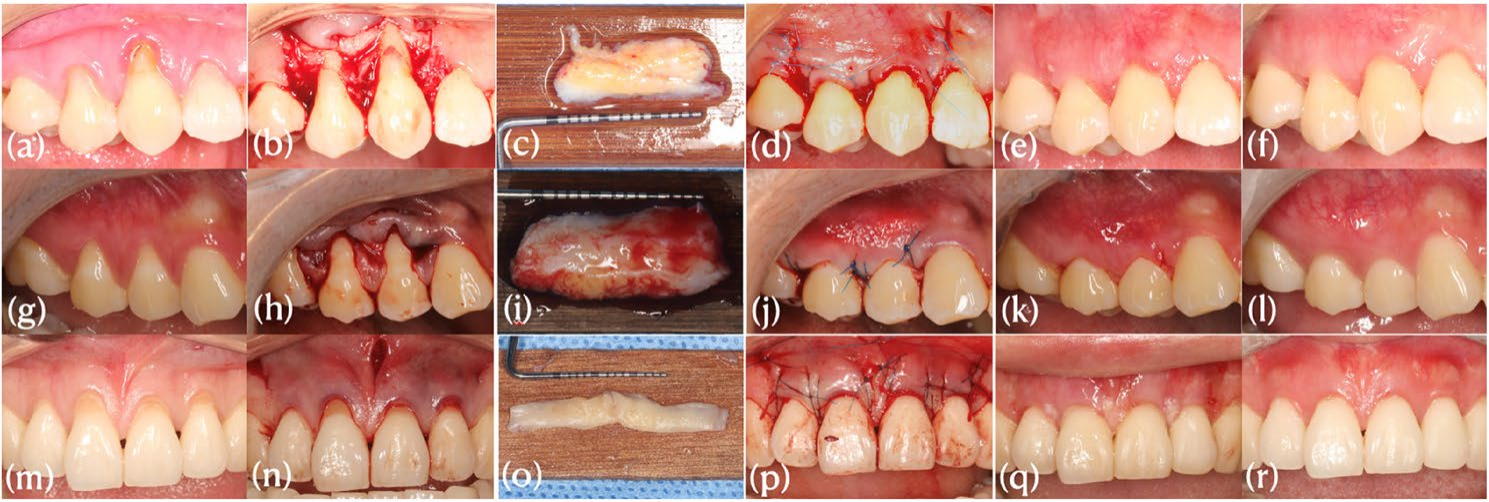
Three RCPs used in this study. (**a-f**) CAF + CTG, (**g-l**) eCAF + CTG, (**m-r**) VISTA + CTG. Each set of images is arranged in the following sequence: baseline, flap preparation, CTG harvesting, suturing, suture removal at 2 weeks post-surgery, and 6 months post-surgery

Three RCPs, namely, CAF [20], eCAF [40], and VISTA [39], were used in this study. In each patient, a CTG was harvested from the ipsilateral maxillary palate, 3 mm apical to the gingival margin (GM) [42]. The CTG was standardized to uniform dimensions of 5 mm in width, 1.0–1.5 mm in thickness, and sufficient length to fully cover the exposed root surfaces. The donor site was protected with a collagen sponge and sutured. The flap was coronally positioned to ensure complete coverage of the CTG by the GM and to achieve maximum root coverage (MRC) [43]. (Fig. 1)

After surgery, the patients were instructed to rinse twice daily with a 0.12% chlorhexidine gluconate solution for 2 weeks. For pain management, ibuprofen (GlaxoSmithKline, Tianjin, China) was prescribed at 500 mg per dose, taken as needed with a minimum interval of 4 h between doses. Systemic antibiotic use after mucogingival surgery remains an open clinical option, but most clinical studies routinely prescribe systemic antibiotics postoperatively [15]. Furthermore, based on relevant literature [16, 22], postoperative medication protocols, and the routine clinical practice of our department, amoxicillin (Zhuhai United Laboratories, Zhongshan, China) was recommended at 500 mg three times daily for 7 consecutive days.

Intraoral sutures were removed at 2 weeks post-surgery. Patients were subsequently scheduled for follow-up visits for clinical evaluations and professional oral hygiene care.

### Clinical assessment and examiner calibration

Plaque index (PLI), bleeding index (BI), probing depth (PD), keratinized gingiva width (KGW), GM location relative to the cementoenamel junction, and clinical attachment level (CAL) of the teeth that underwent RCPs were recorded at baseline and 6 months post-surgery. Surgical technique and duration were documented intraoperatively. At 6 months, periodontal parameters of the teeth that underwent RCPs (PLI, BI, PD, KGW, GM, and CAL) were re-evaluated along with additional parameters: percentage of root coverage (PRC), root coverage esthetic score (RES) [9], and mucosal scar index (MSI) [37]. The primary outcome measures were surgical duration, PRC, RES, and MSI, whereas secondary outcome measures were PLI, BI, PD, KGW, GM, and CAL.

All clinical data were collected by two examiners. To assess intra- and interexaminer reproducibility of the measurements, a calibration process was conducted prior to the study. GM measurements were obtained from 10 distinct GR defects in 10 patients, with duplicate assessments performed at a 1-week interval. Intraexaminer ICCs were 0.934 and 0.921, whereas interexaminer ICCs were 0.828 and 0.897. Calibration was accepted for ICCs > 0.80. Thus, the calibration outcomes for both examiners were deemed acceptable.

### Statistical analysis

Data are presented as mean ± standard deviation (SD) (mean ± SD). The Wilcoxon signed-rank test was used to compare baseline and postoperative values. To account for clustering and intrapatient correlation due to multiple teeth per patient, generalized estimating equations (GEEs) with an exchangeable correlation structure were used for all between-group comparisons. Post hoc multiple comparisons were performed using Bonferroni correction to adjust for multiple testing and control the Type I error rate. GEEs were employed with patient as the clustering unit to adjust for correlation among multiple teeth within the same individual. Age, sex, tooth location, baseline KGW, baseline GM, baseline CAL, and RCP techniques were simultaneously incorporated into the GEE model as covariates to adjust for potential confounding factors. In all analyses, *p* < 0.05 was considered statistically significant. Statistical computations were performed using SPSS 22.0 (IBM, Armonk, USA).

## Results

A total of 304 patients (1,020 teeth) were included in this study (97 males and 207 females), with an average age of 35.07 ± 7.69 years. Of these, 107 teeth were treated with CAF + CTG, 515 with eCAF + CTG, and 398 with VISTA + CTG (Table 1). The PLI, BI, and PD of the affected teeth at baseline and 6 months post-surgery are presented in Table 2.

**Table 1:** Patients’ essential information

	Group A	Group B	Group C	Total
Patient	35	92	177	304
Age	34.89 ± 7.26	36.20 ± 6.90	34.08 ± 8.11	35.07 ± 7.69
Male / Female	11/24	36/56	50/127	97/207
Tooth	81	415	524	1020
CAF / eCAF / VISTA	18/50/13	20/146/249	69/319/136	107/515/398

**Table 2:** PLI, BI, and PD of the patients

			PLI	BI	PD (mm)
Total	Group A	Baseline	0.81 ± 0.72	1.28 ± 0.60	1.15 ± 0.36
		6 months post-surgery	0.95 ± 0.55	1.35 ± 0.76	1.40 ± 0.49
		*p* value	0.1672	0.5385	< 0.0001
	Group B	Baseline	0.91 ± 0.82	1.21 ± 0.63	1.33 ± 0.50
		6 months post-surgery	1.10 ± 0.62	1.11 ± 0.68	1.34 ± 0.50
		*p* value	< 0.0001	0.0008	0.6553
	Group C	Baseline	0.87 ± 0.74	0.94 ± 0.59	1.14 ± 0.34
		6 months post-surgery	0.95 ± 0.68	0.97 ± 0.59	1.06 ± 0.24
		*p* value	0.1004	0.3163	< 0.0001
CAF + CTG	Group A	Baseline	1.28 ± 0.75	1.17 ± 0.38	1.17 ± 0.38
		6 months post-surgery	0.89 ± 0.47	1.28 ± 0.46	1.61 ± 0.50
		*p* value	0.0043	0.3313	0.0018
	Group B	Baseline	1.05 ± 0.83	1.15 ± 0.81	1.35 ± 0.49
		6 months post-surgery	0.72 ± 0.66	0.91 ± 0.55	1.21 ± 0.41
		*p* value	0.0692	0.2044	0.1864
	Group C	Baseline	0.67 ± 0.74	1.06 ± 0.66	1.11 ± 0.32
		6 months post-surgery	0.81 ± 0.71	0.94 ± 0.42	1.03 ± 0.17
		*p* value	0.2605	0.241	0.0572
eCAF + CTG	Group A	Baseline	0.74 ± 0.69	1.44 ± 0.58	1.18 ± 0.39
		6 months post-surgery	1.06 ± 0.55	1.52 ± 0.74	1.34 ± 0.48
		*p* value	0.0279	0.5768	0.0733
	Group B	Baseline	0.82 ± 0.77	1.48 ± 0.65	1.49 ± 0.58
		6 months post-surgery	1.23 ± 0.60	1.51 ± 0.70	1.52 ± 0.57
		*p* value	< 0.0001	0.5734	0.4677
	Group C	Baseline	0.78 ± 0.68	0.85 ± 0.62	1.10 ± 0.31
		6 months post-surgery	0.85 ± 0.69	0.93 ± 0.63	1.08 ± 0.26
		*p* value	0.2236	0.0605	0.0494
VISTA + CTG	Group A	Baseline	0.46 ± 0.52	0.85 ± 0.69	1.00 ± 0.00
		6 months post-surgery	0.62 ± 0.51	0.77 ± 0.93	1.38 ± 0.51
		*p* value	0.1654	0.7762	0.018
	Group B	Baseline	0.95 ± 0.85	1.05 ± 0.54	1.23 ± 0.42
		6 months post-surgery	1.06 ± 0.62	0.89 ± 0.56	1.24 ± 0.43
		*p* value	0.0481	< 0.0001	0.7263
	Group C	Baseline	1.21 ± 0.76	1.09 ± 0.43	1.22 ± 0.42
		6 months post-surgery	1.25 ± 0.54	1.08 ± 0.55	1.04 ± 0.19
		*p* value	0.624	0.9047	< 0.0001

Age, sex, tooth position, baseline KGW and baseline CAL had no statistically significant influence on the primary or secondary outcome measures. Baseline GM had a significant influence on PRC and RES at 6 months post-surgery (*p* < 0.0001). The RCP technique significantly influenced the PRC, RES, MSI, and surgical duration (*p* < 0.05); therefore, separate analyses were conducted for these techniques.

### Clinical results in each group

At baseline, KGW in Groups A, B, and C were 2.15 ± 1.48 mm, 2.06 ± 1.28 mm, and 2.12 ± 1.26 mm, respectively. GM positions were − 2.86 ± 1.42 mm, -2.90 ± 1.34 mm, and − 2.85 ± 1.18 mm, respectively. CAL values were 4.01 ± 1.50 mm, 4.22 ± 1.49 mm, and 3.98 ± 1.26 mm, respectively. At baseline, no significant between-group differences were observed in KGW or GM, whereas CAL in Group C was lower than that in Group B (*p* = 0.0286). Compared with baseline, KGW, GM, and CAL significantly improved in all groups (*p* < 0.0001) (Table 3).

**Table 3:** KGW, GM, and CAL of the patients

			KGW (mm)	GM (mm)	CAL (mm)
Total	Group A	Baseline	2.15 ± 1.48	-2.86 ± 1.42	4.01 ± 1.50
		6 months post-surgery	4.07 ± 2.35	-0.33 ± 0.92	1.74 ± 1.02^γ^
		Δ	1.93 ± 2.03	2.53 ± 1.61	2.27 ± 1.62^γ^
	Group B	Baseline	2.06 ± 1.28	-2.90 ± 1.34	4.22 ± 1.49^β^
		6 months post-surgery	4.37 ± 1.12^β^	-0.14 ± 0.44	1.47 ± 0.76^β^
		Δ	2.31 ± 1.43	2.86 ± 1.33	2.75 ± 1.40
	Group C	Baseline	2.12 ± 1.26	-2.85 ± 1.18	3.98 ± 1.26
		6 months post-surgery	4.18 ± 0.92	-0.15 ± 0.37	1.20 ± 0.42
		Δ	2.06 ± 1.38	2.74 ± 1.16	2.78 ± 1.20
CAF + CTG	Group A	Baseline	3.22 ± 1.96^α, γ^	-3.44 ± 1.79	4.61 ± 2.06
		6 months post-surgery	6.28 ± 3.54	-0.33 ± 0.97	1.94 ± 1.21^γ^
		Δ	3.06 ± 2.21	3.11 ± 1.52	2.67 ± 1.33
	Group B	Baseline	1.54 ± 1.19	-3.06 ± 1.79	4.41 ± 2.19
		6 months post-surgery	4.22 ± 1.11	-0.20 ± 0.70	1.40 ± 0.94
		Δ	2.65 ± 1.27	2.85 ± 1.63	3.01 ± 1.83
	Group C	Baseline	1.48 ± 1.05	-3.05 ± 1.22	4.17 ± 1.39
		6 months post-surgery	4.09 ± 0.92	-0.21 ± 0.42	1.25 ± 0.43
		Δ	2.61 ± 1.32	2.96 ± 1.22	2.92 ± 1.42
eCAF + CTG	Group A	Baseline	1.78 ± 0.93^α, γ^	-2.62 ± 1.31	3.80 ± 1.31
		6 months post-surgery	3.12 ± 1.22^α, γ^	-0.42 ± 1.00	1.76 ± 1.04^γ^
		Δ	1.34 ± 1.88	2.20 ± 1.69^γ^	2.04 ± 1.80^γ^
	Group B	Baseline	2.27 ± 1.28	-2.76 ± 1.01	4.25 ± 1.06^β^
		6 months post-surgery	4.21 ± 1.33	-0.09 ± 0.34	1.62 ± 0.73^β^
		Δ	1.93 ± 1.48	2.66 ± 0.96	2.63 ± 1.05
	Group C	Baseline	2.23 ± 1.22	-2.80 ± 1.03	3.90 ± 1.07
		6 months post-surgery	4.19 ± 0.94	-0.15 ± 0.39	1.23 ± 0.44
		Δ	1.96 ± 1.34	2.64 ± 0.98	2.67 ± 1.01
VISTA + CTG	Group A	Baseline	2.08 ± 1.85	-3.00 ± 1.08	4.00 ± 1.08
		6 months post-surgery	4.69 ± 1.18	-0.08 ± 0.28	1.38 ± 0.51
		Δ	2.62 ± 1.50	2.92 ± 1.12	2.62 ± 1.12
	Group B	Baseline	1.98 ± 1.27^β^	-2.97 ± 1.46	4.20 ± 1.63
		6 months post-surgery	4.48 ± 0.98^β^	-0.15 ± 0.47	1.40 ± 0.74^β^
		Δ	2.51 ± 1.38^β^	2.81 ± 1.41	2.80 ± 1.53
	Group C	Baseline	2.20 ± 1.35	-2.86 ± 1.46	4.08 ± 1.57
		6 months post-surgery	4.20 ± 0.85	-0.09 ± 0.28	1.13 ± 0.33
		Δ	2.01 ± 1.43	2.77 ± 1.39	2.96 ± 1.46

* Within each subgroup, significant differences were observed in KGW, GM, and CAL between baseline and 6 months post-surgery (*p* < 0.0001)

Δ represents the difference between baseline and 6 months post-surgery

Significant difference was observed between Group A and B (*p* < 0.05). β: Significant difference was observed between Group B and C (*p* < 0.05). γ: Significant difference was observed between Group A and C (*p* < 0.05)

**Fig. 2:**
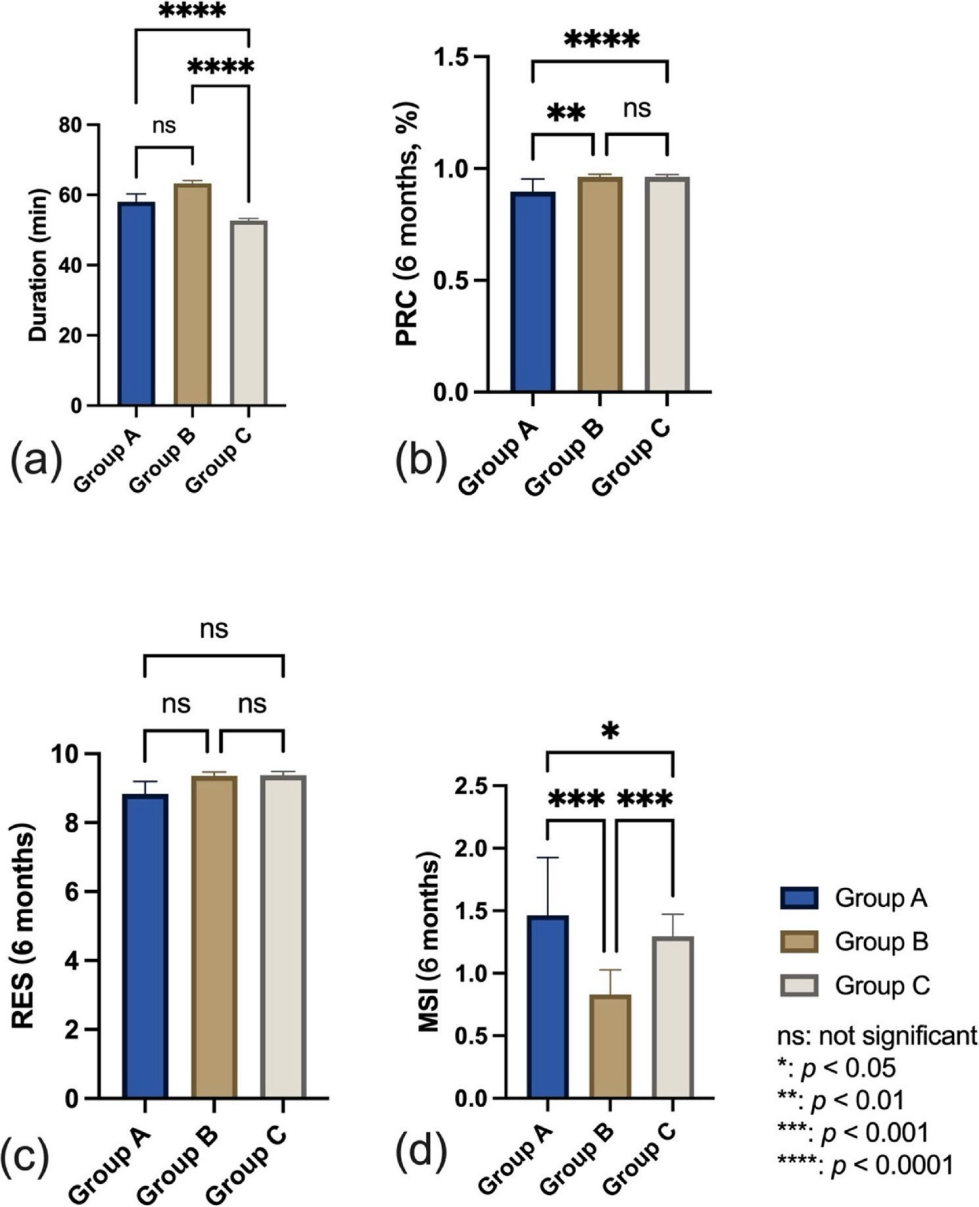
Surgical duration, PRC, RES, and MSI in each group. Data are presented as adjusted means ± 95% CI derived from GEE model. **a** Surgical duration in each group, **b** PRC at 6 months post-surgery in each group, **c** RES values at 6 months post-surgery in each group, **d** MSI values at 6 months post-surgery in each group

Post-surgery CAL in Group C (1.20 ± 0.42 mm) was significantly lower than that in Group A (1.74 ± 1.02 mm) and Group B (1.47 ± 0.76 mm) (*p* < 0.0001) (Table 3). At 6 months post-surgery, PRC in Group A (89.71 ± 25.18%) was significantly lower than that in Group B (96.34 ± 11.91%, *p* = 0.0117) and Group C (96.34 ± 10.95%, *p* < 0.0001). No significant differences in RES were observed among the three groups (*p* > 0.05). The surgical duration in Group C was significantly shorter than that in the other two groups (*p* < 0.0001) (Table 4; Fig. 2).

**Table 4:** Surgical duration, PRC, RES, MSI of the patients

		Duration (min)	PRC (6 m, %)	RES (6 m)	MSI (6 m)
Total	Group A	58.09 ± 10.10^γ^ (55.81, 60.28)	89.71 ± 25.18^α, γ^ (84.14, 95.28)	8.85 ± 1.60 (8.47, 9.20)	1.46 ± 2.10^α, γ^ (1.00, 1.93)
	Group B	63.30 ± 8.16^β^ (62.52, 64.09)	96.34 ± 11.91 (95.19, 97.49)	9.36 ± 1.10 (9.26, 9.47)	0.83 ± 2.04^β^ (0.63,1.03)
	Group C	52.69 ± 7.03 (52.08, 53.29)	96.34 ± 10.95 (95.40, 97.28)	9.38 ± 1.21 (9.28, 9.49)	1.30 ± 2.04 (1.12, 1.47)
CAF + CTG	Group A	58.17 ± 6.84^α, γ^ (54.76, 61.57)	94.44 ± 16.17 (86.40, 102.50)	8.94 ± 1.21 (8.29, 9.58)	3.56 ± 1.61^α, γ^ (2.76, 4.36)
	Group B	48.70 ± 2.34^β^ (47.60, 49.80)	96.25 ± 12.23 (90.52, 102.01)	9.56 ± 1.09 (9.04, 10.06)	0.97 ± 1.96 (0.03, 1.92)
	Group C	48.74 ± 3.81 (47.82, 49.66)	100.00 ± 0.00 (100.00, 100.00)	9.08 ± 1.26 (8.78, 9.38)	0.38 ± 1.14 (0.10, 0.65)
eCAF + CTG	Group A	55.08 ± 10.29 (52.16, 58.00)	85.33 ± 28.81^α^ (76.86, 93.81)	8.54 ± 1.82 (8.02, 9.06)	0.91 ± 1.98^α^ (0.35, 1.47)
	Group B	57.63 ± 6.17^β^ (56.62, 58.64)	97.43 ± 9.00 (95.96, 98.90)	9.17 ± 1.17 (8.98, 9.36)	1.39 ± 2.62 (0.96, 1.82)
	Group C	51.04 ± 4.83 (50.51, 51.57)	94.93 ± 13.03 (93.50, 96.37)	9.38 ± 1.14 (9.25, 9.50)	1.71 ± 2.16 (1.48, 1.95)
VISTA + CTG	Group A	69.31 ± 2.87 (67.57, 71.04)	97.44 ± 9.25 (91.85, 103.32)	9.85 ± 0.32 (9.66, 10.04)	0.69 ± 1.11 (0.02, 1.36)
	Group B	67.80 ± 5.66^β^ (67.10, 68.51)	95.71 ± 13.31 (94.04, 97.37)	9.46 ± 1.04 (9.33, 9.59)	0.49 ± 1.54 (0.30, 0.69)
	Group C	58.56 ± 8.93 (57.04, 60.07)	97.79 ± 7.12 (96.59, 99.00)	9.54 ± 1.32 (9.32, 9.77)	0.78 ± 1.86 (0.46, 1.10)

*All data were presented as mean ± SD with 95% confidence interval (95%CI)

α: Significant difference was observed between Group A and B (*p* < 0.05). β: Significant difference was observed between Group B and C (*p* < 0.05). γ: Significant difference was observed between Group A and C (*p* < 0.05)

### Clinical results of patients who underwent CAF + CTG

**Fig. 3:**
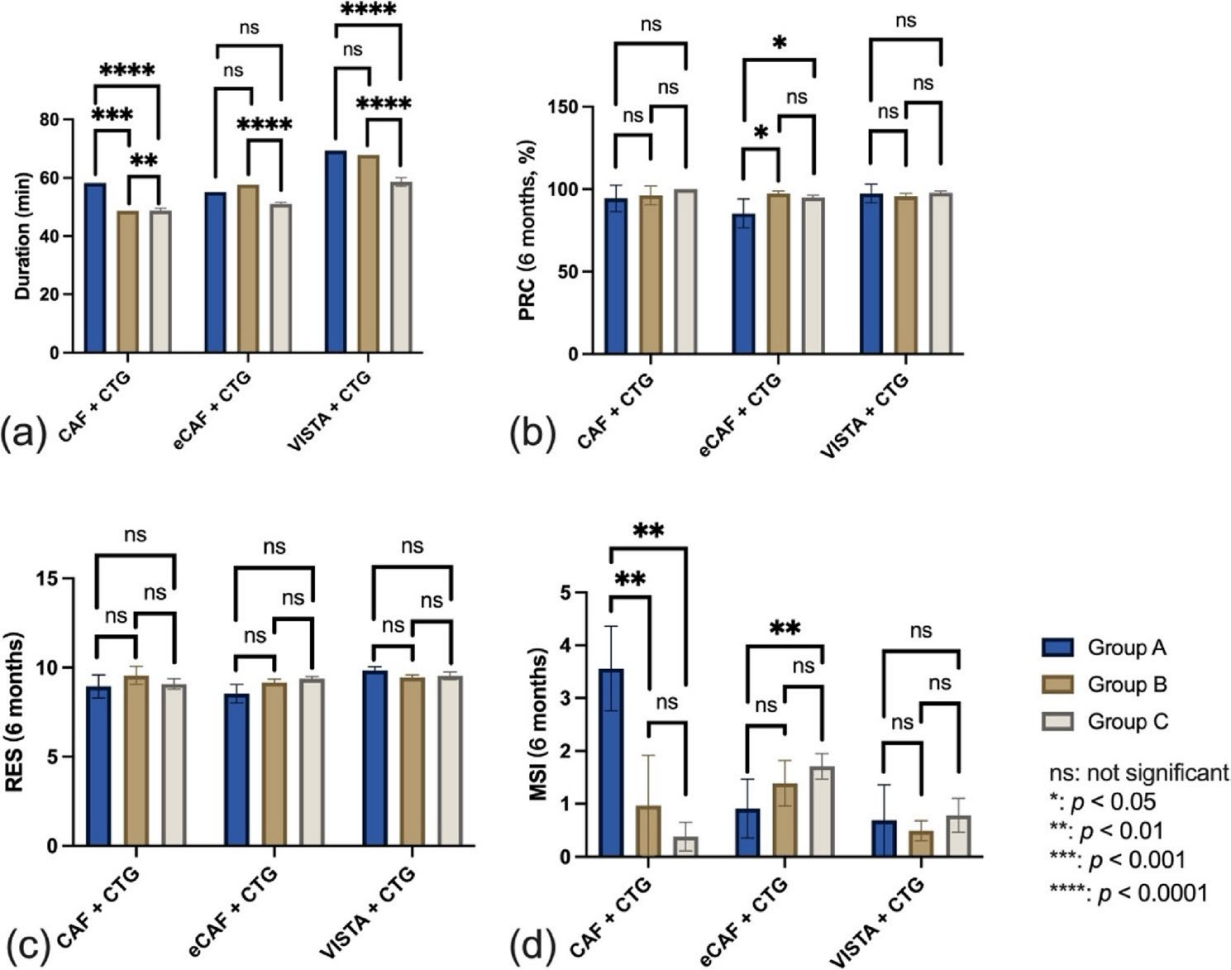
Surgical duration, PRC, RES, and MSI in patients treated with different RCPs. Data are presented as adjusted means ± 95% CI derived from GEE model. **a** Surgical duration of the patients who underwent RCPs, **b** PRC of the patients at 6 months post-surgery, **c** RES values of the patients at 6 months post-surgery, **d** MSI values of the patients at 6 months post-surgery

At 6 months post-surgery, CAL in Group C (1.25 ± 0.43 mm) was significantly lower than that in Group A (1.94 ± 1.21 mm) (*p* = 0.0097). No significant differences were observed in KGW, GM, ΔKGW, ΔGM, or ΔCAL among the three groups (*p* > 0.05) (Table 3). No significant differences were observed in PRC or RES among the groups (*p* > 0.05). MSI in Group A (3.56 ± 1.61) was significantly higher than that in Group B (0.97 ± 1.96) (*p* = 0.0024) and Group C (0.38 ± 1.14) (*p* = 0.0012) (Table 4; Fig. 3). Surgical duration in Group A was significantly longer than that in Group B (*p* = 0.0001) and Group C (*p* < 0.0001) (Fig. 3).

### Clinical results of patients who underwent eCAF + CTG

At 6 months post-surgery after eCAF + CTG, significant differences were observed in CAL (*p* = 0.0009) and ΔCAL (*p* = 0.0033) between Group A and Group C (Table 3). The PRC in Group A (85.33 ± 28.81%) was significantly lower than that in Group B (97.43 ± 9.00%, *p* = 0.0124) and Group C (94.93 ± 13.03%, *p* = 0.0233) (Table 4; Fig. 3). The surgical duration in Group C was significantly shorter than that in Group B (*p* < 0.0001) (Table 4; Fig. 3).

### Clinical results of patients who underwent VISTA + CTG

At 6 months post-surgery following VISTA + CTG, both KGW (*p* = 0.0003) and CAL (*p* < 0.0001) were significantly lower in Group C than in Group B (Table 3). No significant differences were observed in PRC, RES, or MSI among the three groups (*p* > 0.05). The surgical duration in Group C was significantly shorter than that in Group B (*p* < 0.0001) (Table 4; Fig. 3).

## Discussion

Surgeon experience level may have a measurable influence on surgical outcomes. However, RCPs are already established as a mature surgical approach for treating GR, and their efficacy and stability have been validated by multiple clinical studies [1, 11, 35].

The parameters employed to assess the efficacy of RCPs include both clinical periodontal indicators and esthetic-related indicators. Clinical periodontal indicators include PLI, BI, PD, KGW, GM, CAL, and PRC. PLI, BI, and PD reflect patients’ oral hygiene status and gingival health. Although significant differences in the pre- and postsurgical values of PLI, BI, and PD were observed in some subgroups, all values remained within periodontally healthy ranges [25]. An appropriate KGW is crucial for the health of periodontal tissues [10, 30]. In this study, KGW in all groups increased significantly at 6 months post-surgery compared with baseline (*p* < 0.0001). After RCPs, PD generally does not undergo significant change. However, owing to coronal advancement of the GM and formation of new attachment on the root surface, CAL decreases [17]. In this study, both GM and CAL significantly improved in all groups at 6 months post-surgery compared to baseline (*p* < 0.0001). Moreover, postsurgical CAL in Group C is significantly lower than in the other two groups. The PRC reported in previous studies across various RCPs generally exceeds 90% [1, 16, 29]. In this study, PRC in Group A (89.71 ± 25.18%) was significantly lower than in Group B (96.34 ± 11.91%, *p* = 0.0117) and Group C (96.34 ± 10.95%, *p* < 0.0001), indicating that surgeon experience level positively influences PRC. The esthetic indicators selected in this study include RES and MSI. A higher RES indicates better postsurgical esthetic results [9]. Previous studies have demonstrated a correlation between RES and PRC, with a higher PRC associated with greater RES [8]. In this study, RES values for Groups A, B, and C were 8.85 ± 1.60, 9.36 ± 1.10, and 9.38 ± 1.21, respectively, with no significant differences among the three groups, suggesting that surgeon experience level has little effect on RES. MSI is an index proposed by Wessels et al. [37] to assess postoperative mucosal scar formation. Compared with RES, MSI places greater emphasis on assessing scar shape and size. In this study, MSI in Group A was significantly higher than in Group B (*p* = 0.0001) and Group C (*p* = 0.0231), indicating that surgeon experience level may influence scar formation. However, MSI in Group B was significantly lower than in Group C (*p* = 0.0003), which may be attributed to senior surgeons’ tendency to select patients with a broader range of surgical indications and more challenging cases. Therefore, while surgeon experience does not significantly affect overall esthetic outcomes.

A prolonged surgical duration may increase patient discomfort. We observed that surgical duration in Group C was significantly shorter than in the other two groups (*p* < 0.0001). Among patients treated with CAF + CTG, significant differences existed among the three groups. For patients treated with eCAF + CTG and VISTA + CTG, surgical duration in Group C was significantly shorter than in Group B (*p* < 0.0001) and numerically shorter than in Group A. Therefore, surgeon experience contributes to reducing surgical duration. Although prolonged surgical duration does not significantly affect long-term prognosis of RCPs, shortening surgical time is beneficial in alleviating patients’ intraoperative discomfort [10].

CAF + CTG is considered the gold standard for treating GR [5], with reported PRC ranging from 89.4% to 100% [4, 13, 29]. In CAF, mesial and distal vertical incisions improve the surgical field of view, reduce the difficulty of gingival flap elevation, and facilitate the loosening of gingival flap tension. Among patients who underwent CAF + CTG in this study, the PRC values in Groups A and B were approximately 95%, the PRC in Group C approached 100%, indicating that a satisfactory PRC could be achieved in all three groups. No significant differences in KGW or GM were observed among the three groups at 6 months post-surgery. In addition, no statistically significant difference was observed in RES among the three groups at 6 months post-surgery. However, MSI at 6 months post-surgery in Group A was significantly higher than that in the other two groups (*p*_A to B_ = 0.0024, *p*_A to C_ = 0.0127). We speculate that this may be attributed to the fact that less experienced surgeons often have difficulty achieving precise suturing of vertical incisions, potentially leading to increased postoperative scar formation [41]. Nevertheless, we believe that surgeon experience generally has no significant effect on the clinical and esthetic outcomes of CAF + CTG.

The eCAF technique avoids vertical incisions by extending the incision mesially and distally, thereby reducing the risk of scar formation associated with vertical incisions [41]. Tension control in the eCAF technique typically involves inserting the blade at a specific angle into the inner aspect of the gingival flap to sequentially sever the superficial and deep submucosal connective tissues [40]. Chen et al. [14] demonstrated that, compared to the trapezoidal CAF and TUN techniques, the eCAF technique presents higher rates of complete root coverage and RES. In this study. The PRC among patients who underwent eCAF + CTG in Group A was 85.33 ± 28.81%, while the PRC values in the other subgroups were all greater than 90%. This may be attributed to the absence of vertical incisions, which obstructs the surgeon’s visual field, thereby impeding less experienced surgeons from effectively managing flap tension, consequently compromising the ultimate PRC. However, such explanations remain hypothetical due to the lack of relevant quantitative indicators. As for the esthetic outcomes of eCAF + CTG, there was no significant difference in RES among the three groups. The MSI of Group A is significantly lower than that of Group C (*p* = 0.0031). Meanwhile, postsurgical CAL in Group C was significantly lower than that in the other two groups (*p*_A to C_ = 0.0009; *p*_B to C_ < 0.0001). Therefore, surgeon experience level does not significantly affect esthetic outcomes of eCAF + CTG, but may influence the ultimate PRC. We recommend that less experienced surgeons postpone adopting eCAF until they have accumulated sufficient experience.

The VISTA technique, which modifies the tunneling approach by incorporating a vertical incision in the vestibular sulcus for flap preparation while avoiding damage to the interdental papilla, thereby reducing surgical trauma and enhancing clinical outcomes [16]. Among patients who underwent VISTA + CTG, no significant differences were observed in PRC, RES, or MSI among the three groups at 6 months post-surgery. However, we do not recommend that less experienced surgeons adopt VISTA as the preferred technique for root coverage. VISTA requires greater caution and focus from surgeons than CAF and eCAF. Nevertheless, this caution and focus exhibited by less experienced surgeons may prolong surgical duration and potentially exacerbate patient discomfort. In this study, the mean surgical duration in Group A was 69.31 ± 2.87 min in the VISTA + CTG group and 58.17 ± 6.84 min in the CAF + CTG group. Given these findings, we conclude that while VISTA + CTG offers minimal trauma and predictable surgical outcomes, it is more suitable for surgeons with considerable experience as the preferred technique for root coverage.

The results of the GEE analysis show that baseline GM has a statistically significant influence on PRC and RES at 6 months post-surgery (*p* < 0.0001), consistent with the findings of Domenico et al. [21]. In the present study, no significant differences in baseline GM were observed among the groups, indicating that baseline GM did not affect the results.

This study has the following limitations. This retrospective study lacked randomization and was prone to operator selection bias. Since observations were made at the tooth level while grouping was based on patients, GEEs were used for statistical adjustment. The number of patients in Group A did not match that of the other two groups, a difference related to the surgeons’ years of experience. In addition, sample size calculations and power analyses were not performed a priori. This was primarily due to the retrospective nature of the study and insufficient preliminary data. Smoking is among the factors affecting surgical outcomes; therefore, we excluded heavy smokers (> 10 cigarettes per day). However, daily cigarette consumption was not recorded for each patient in this study, which represents an additional limitation.

## Conclusions

Surgeons with varying levels of experience can achieve favorable root coverage outcomes using different techniques. Surgeon experience level is positively associated with PRC in RCPs. Although esthetic outcomes remain comparable across experience levels, more experienced surgeons tend to have shorter surgical durations. CAF + CTG is recommended as the preferred technique for less experienced surgeons. For surgeons with substantial clinical experience, eCAF + CTG and VISTA + CTG are recommended for treating GR.

## Data Availability

The datasets used and/or analysed during the current study are available from the corresponding author on reasonable request.

## Abbreviations

GR: Gingival recession
RCPs: Root coverage procedures
CTG: Connective tissue graft
CAF: Coronally advanced flap
eCAF: Envelope coronally advanced flap
VISTA: Vestibular incision subperiosteal tunnel access
GM: Gingival margin
MRC: Maximum root coverage
PLI: Plaque index
BI: Bleeding index
PD: Probing depth
KGW: Keratinized gingiva width
CAL: Clinical attachment level
PRC: Percentage of root coverage
RES: Root coverage esthetic score
MSI: Mucosal scar index
GEE: Generalized estimating equations
